# Comparison of Rebound Tonometry, Perkins Applanation Tonometry and Ocular Response Analyser in Mucopolysaccharidosis Patients

**DOI:** 10.1371/journal.pone.0133586

**Published:** 2015-08-28

**Authors:** Joanna Wasielica-Poslednik, Christina Butsch, Christina Lampe, Heike Elflein, Julia Lamparter, Veronika Weyer, Susanne Pitz

**Affiliations:** 1 Department of Ophthalmology, University Medical Center of the Johannes Gutenberg- University Mainz, Mainz, Germany; 2 Department of Pediatrics, Dr. Horst Schmidt Clinic GmbH, Wiesbaden, Germany; 3 Institute for Medical Biostatistics, Epidemiology and Informatics (IMBEI), University Medical Center of the Johannes Gutenberg University Mainz, Mainz, Germany; Bascom Palmer Eye Institute, University of Miami School of Medicine;, UNITED STATES

## Abstract

**Aims:**

To investigate the feasibility and to compare three devices measuring intraocular pressure (IOP) in mucopolysaccharidosis patients (MPS): iCare rebound tonometer (RT), Perkins applanation tonometer (PAT) and ocular response analyzer (ORA)

**Methods:**

MPS patients who underwent at least two examinations out of: RT, PAT and ORA at the same visit were identified and retrospectively analyzed in this study.

**Results:**

17 patients fulfilled the inclusion criterion. In all 17 patients IOP measurements were performed with RT (34 eyes) and ORA (33 eyes), while PAT measurement was possible in only 12 (24 eyes) patients. The RT, corneal-compensated intraocular pressure (IOPcc) and Goldmann-correlated intraocular pressure (IOPg) differed relevantly from IOP assessed with PAT. Corneal clouding in MPS patients correlated positively with PAT, RT and IOPg (r = 0.3, 0.5, and 0.5 respectively), but not with IOPcc (r = 0.07). The MPS-related corneal clouding correlated positively with biomechanical corneal parameters assessed with ORA: corneal hysteresis (r = 0.77) and corneal resistance factor (r = 0.77) either.

**Conclusions:**

RT and ORA measurements were tolerated better than applanation tonometry in MPS patients. IOP measurements assessed with RT and ORA differed relevantly from PAT. Corneal-compensated IOP assessed with ORA seems to be less affected by the MPS-related corneal clouding than applanation or rebound tonometry. RT and ORA measurements should be preferred for IOP assessment in patients with MPS.

## Introduction

The Mucopolysaccharidoses (MPS) represent a heterogeneous group of rare lysosomal storage disorders, caused by deficiency of enzymes catalyzing glycosaminoglycans (GAG). With the cumulative rates for all MPS types of approximately 3,4–4,5 in 100.000 live births, MPS belong to the orphan diseases [1]. Deficiency of GAG-catalyzing enzymes with consequent intralysosomal accumulation of interstage products results in seven different MPS types characterized by chronic and progressive course of the disease as well as reduced life expectancy [2]. MPS patients suffer from clinical abnormalities in multiple organs. The most common ocular manifestations are: corneal clouding, ocular hypertension/glaucoma, retinal degeneration and optic nerve swelling or atrophy. Progressive pseudoexophthalmos, hypertelorism, strabismus and farsightedness have also been reported [3].

The prevalence of glaucoma in MPS patients was reported to be as high as 6,8% and occurs most commonly in MPS type I, IV and VI [4,5]. The main pathogenic mechanism underlying glaucoma in MPS patients is thought to be the obstruction of the trabecular meshwork resulting in an impaired outflow of aqueous humor [6]. A further mechanism is supposed to derive from the narrowing of the anterior chamber angle due to increased thickness of the iris and peripheral cornea secondary to GAG accumulation [7].

To date, the reduction of IOP represents the major therapeutic approach in the management of glaucoma [8]. Thus, an accurate and reliable IOP measurement is indispensable for diagnosis and follow-up of glaucoma.

The accurate diagnosis of glaucoma in MPS patients is hampered by a number of physical and psychological factors [4,9]. One is the corneal clouding, which impairs the visualization of the optic nerve and its potential glaucomatous cupping as well as proper gonioscopic assessment of anterior chamber angle. Clinical value of visual field testing in MPS patients may be also reduced due to media opacification/decreased visual acuity or poor cooperation determined by young age, mental status or short stature, not allowing a comfortable positioning during examination. The same factors are critical for other ophthalmological examinations such as slit lamp investigation or applanation tonometry. Thus, spectrum and quality of examinations to rule out glaucoma performed in MPS patients is often far from the routinely practiced standard of care.

We analyzed IOP values assessed with rebound tonometry (RT), ocular response analyzer (ORA, Reichert Inc., Buffalo, NY) and hand-held Perkins applanation tonometer (PAT) in order to potentially optimize IOP assessment in MPS patients. We sought to compare non-invasive and thus potentially better tolerated IOP-measuring devices such as RT and ORA to gold standard applanation tonometry as represented by PAT in MPS patients.

## Methods

We analyzed charts of MPS patients examined in the outpatient clinic at the Department of Ophthalmology of the University Medical Center, Johannes Gutenberg University, Mainz, Germany in 2011. The retrospective medical chart review was approved by the Ethics committee of Rhineland Palatinate, Germany. Informed consent was not required. The patients´ charts were anonymized prior to analysis.

For further evaluation we identified patients, who underwent at least two examinations out of RT, PAT and ORA measurements during the same visit.

All MPS patients were transferred from the Department for Lysosomal Storage Disorders of the Children´s Hospital, Mainz University Medical Center and MPS was confirmed by molecular genetic studies.

The PAT is based on Goldmann principles and utilizing the Goldmann disposable prisms. The measurement of IOP with PAT requires instilling oxybuprocain-HCl/fluorescein-Na (Thilorbin) eye drops in the lower conjunctival cul-de-sac in both eyes and was performed without pupil dilatation in all cases.

The ORA utilizes a visco-elastic structure of the human corneal tissue in a dynamic bi-directional applanation process. The difference in inward and outward pressure values is called corneal hysteresis (CH) and the average of both values provides Goldmann-correlated intraocular pressure (IOPg). Calculated on the basis of the measured CH, the ORA provides two other parameters: CRF (corneal rigidity) and corneal compensated intraocular pressure (IOPcc), which has been reported to be less affected by corneal properties such as CCT than other tonometry readings [10].

The iCare RT is a portable, self-calibrating tonometer housing a round tipped probe of 0.9 mm radius, held in position by an electromagnetic field. The probe collides with the central cornea, inducing a small induction which is followed by the measurement of the impact duration. The probe rebounds faster with increased IOP. Measurements are taken within 0.1 seconds. RT allows multiple, rapid IOP-measurements with no need of corneal anaesthesia [11].

Grading of corneal clouding was done according to Couprie et al. [12] as follows: grade 1 –no corneal clouding visible; grade 2 –mild corneal clouding, still allowing good visibility of details of the anterior chamber, iris and retina; grade 3 –moderate corneal clouding with partial masking of anterior chamber and iris details as well as reduced fundus view; grade 4 –severe corneal clouding without view on anterior chamber and posterior chamber of the eye.

## Statistics

For descriptive analysis concerning the continuous and not normally distributed values, the median, 25% and 75% percentiles, minimum and maximum values were calculated.

The Friedman test was performed for comparing the IOP values measured by RT and ORA (IOPcc and IOPg) with the gold standard–PAT-IOP- separately for the right and left eyes.

For especially evaluating the concordance of the different methods intraclass correlation coefficients (ICCs) were calculated and Bland-Altman plots are presented.

Spearmann´s correlation coefficient was used to assess correlation between corneal clouding and RT-IOP, PAT-IOP as well as ORA parameters.

As this is an explorative study, no adjustments for multiple testing were done. The statistical tests were performed for illustrative purposes rather than for hypothesis testing. P-values are given for descriptive reasons only and therefore should be interpreted with caution.

All statistical analyses were performed using SPSS 21 and R version 3.1.3

## Results

17 MPS patients (8 females and 9 males aged mean 24.0 ± 10.8, range 9–49 years, Table 1) were enrolled in this study. This group consisted of 3 MPS I, 3 MPS II, 5 MPS IV, 6 MPS VI patients. All MPS I, II and VI patients were treated with enzyme replacement therapy.

**Table 1:** Clinical details regarding study patients. Grading of corneal clouding according to Couprie et al. [12]; BCVA–best corrected visual acuity (decimal); OD–right eye; OS–left eye.

No.	MPS type	Age	Eye	corneal clouding	BCVA	Other
1.	I	31,4	OD	1	1	
			OS	1	1	
2.	I	38,2	OD	3	0,4	
			OS	3	0,5	
3.	I	36,4	OD	4	Hand-motion	
			OS	1	0,5	corneal transplantation*
4.	II	20,8	OD	1	0,5	papilloedema
			OS	1	0,25	papilloedema
5.	II	30,6	OD	1	0,63	tapetoretinal degeneration
			OS	1	0,63	tapetoretinal degeneration
6.	II	49,7	OD	1	o,4	tapetoretinal degeneration
			OS	1	0,25	tapetoretinal degeneration
7.	IV	12	OD	2	0,63	
			OS	2	0,8	
8.	IV	25,1	OD	2	0,63	
			OS	2	1	
9.	IV	9,3	OD	2	1	
			OS	2	1	
10.	IV	19,2	OD	2	N/A	
			OS	2	N/A	
11.	IV	25	OD	2	1,25	
			OS	2	1	
12.	VI	28,7	OD	2	0,8	
			OS	2	0,8	
13.	VI	9,2	OD	2	0,5	
			OS	3	0,5	
14.	VI	17,2	OD	2	light perception	non-glaucomatous optic atrophy
			OS	2	nil light perception	non-glaucomatous optic atrophy
15.	VI	18,2	OD	3	0,32	
			OS	3	0,32	
16.	VI	30,7	OD	2	1	
			OS	2	1,25	
17.	VI	23	OD	1	0,8	
			OS	1	1	

*Left eye of the patient no. 3 was excluded from the statistical analysis due to corneal transplantation.

Visual acuity and grade of corneal clouding as well as additional clinical information collected from patients´ charts are shown in Table 1.

In our cohort of 17 MPS patients 34 eyes were measured with RT and 24 eyes with PAT. In 5 patients PAT could not be performed due to reduced compliance (3 pediatric patients) or was refused due to the necessity of application of the anesthetic eye drops (2 adult patients). In 33 eyes CH, CRF, IOPg and IOPcc were obtained using ORA. The measurement with ORA of one left eye was not possible due to the proximity sensor measurement termination. Goldmann applanation tonometry (GAT) could not be performed in our study cohort due to the young age and/or MPS-related symptoms (e.g. short stature).

**Fig 1:**
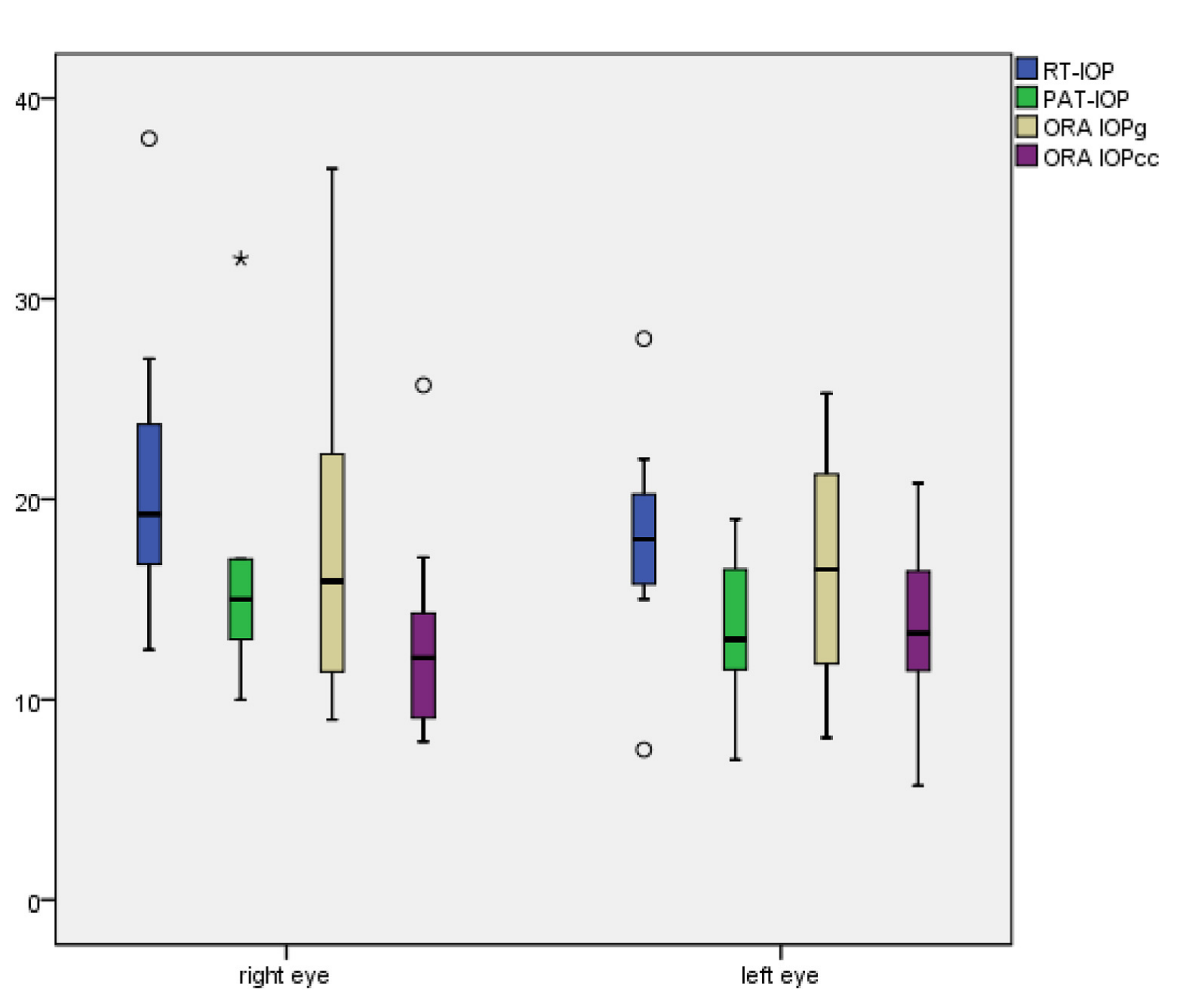
X-axis: right and left eyes; Y-axis: median values and ranges of rebound tonometry (RT-IOP), Perkins applanation tonometry (PAT-IOP), Goldmann-correlated intraocular pressure (ORA-IOPg) and corneal-compensated intraocular pressure (ORA-IOPcc) in mmHg.

Median, 25% percentile, 75% percentile and ranges of RT-IOP, PAT-IOP, IOPcc, IOPg, CH and CRF for right and left eyes are shown in Table 2 and graphically presented in Fig 1 (median IOP values).

**Table 2:** Median, 25% percentile, 75% percentile and ranges of rebound tonometry (RT-IOP), Perkins applanation tonometry (PAT-IOP), corneal-compensated intraocular pressure (IOPcc), Goldmann-correlated intraocular pressure (IOPg), corneal hysteresis (CH) and corneal resistance factor (CRF) for the right (OD) and left eyes (OS).

	OD median	OD 25% percentile	OD 75% percentile	OD Range	OS median	OS 25% percentile	OS 75% percentile	OS Range
**RT-IOP**	19	14,5	23,8	7,5–38,0	17,8	15,6	20,4	13–28
**IOPcc**	13	10	14,1	7,9–25,7	15,2	11,9	17,1	5,7–20,8
**IOPg**	16,1	12,1	19,3	9–36,5	18,9	12,6	21,2	10,1–25,3
**PAT-IOP**	15	13	17	10,0–32,0	13	12	17	11–19
**CH**	14	11	16,9	10,2–28,3	13,6	11,3	15	9,7–19,7
**CRF**	13,4	11,1	16,2	8,6–31,9	14,2	10,4	15,2	9,4–20,0

All IOP values are presented in mmHg.

**Fig 2:**
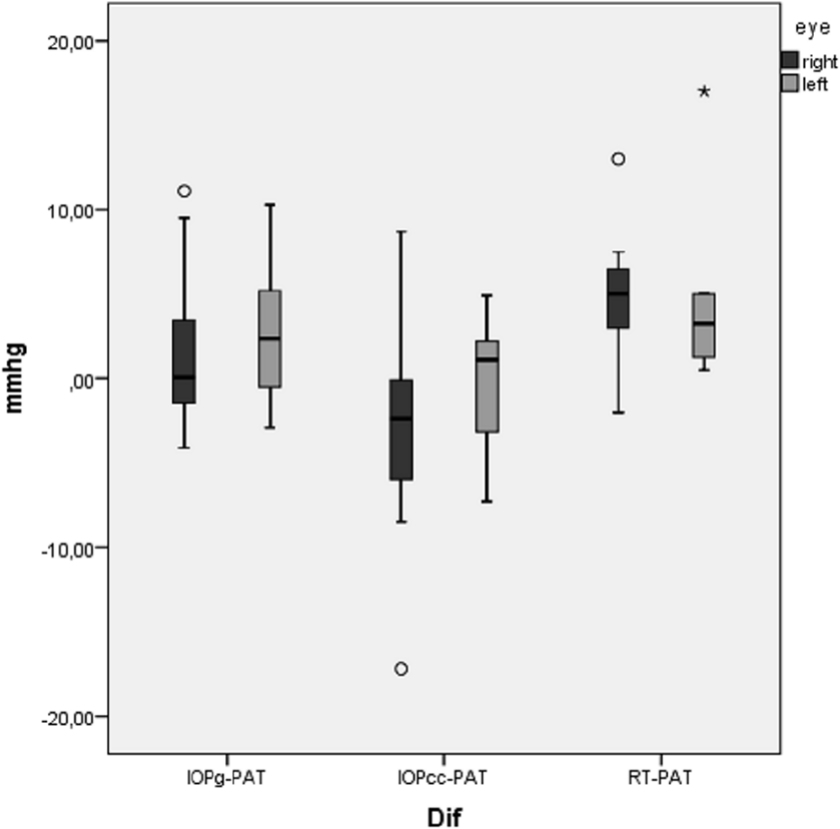
X-axis: eye 1 –right eye; eye 2 –left eye; Y-axis: median values and ranges of the differences between Goldmann-correlated intraocular pressure and Perkins applanation tonometry (ΔIOPg-PAT); between corneal-compensated intraocular pressure and PAT (ΔIOPcc-PAT) and between rebound tonometry and PAT (ΔRT-PAT) in mmHg.

The statistical analysis with Friedman test revealed statistically relevant differences (p = 0.002 for the right eyes and 0,003 for the left eyes) between PAT-IOP and other tonometry readings (IOPg, IOPcc and RT-IOP) in MPS patients (Fig 2).

ICCs are presented in Table 3. The agreement was very good between RT and PAT for the right eyes; good between IOPg and PAT in the right eyes; moderate between IOPg and PAT and between IOPcc and PAT in the left eyes; weak between RT and PAT and between IOPcc and PAT in the left eyes.

**Table 3:** Intraclass correlation coefficients (ICCs), 95% confidence intervals and p-values for agreement between: rebound tonometry (RT-IOP), Goldmann-correlated intraocular pressure (ORA-IOPg), corneal-compensated intraocular pressure (ORA-IOPcc) and Perkins applanation tonometry (PAT-IOP) in right (OD) and left (OS) eyes.

	ICC	95% confidence interval	p-value
**RT-PAT-OD**	0.805	0.454 0.94	<0.001
**RT-PAT-OS**	0.310	(-0,439) 0.687	0.284
**IOPg-PAT-OD**	0.760	0.357 0.924	0.001
**IOPg-PAT-OS**	0.498	(-0.149) 0.846	0.06
**IOPcc-PAT-OD**	0.291	(-0.312) 0.727	0.168
**IOPcc-PAT-OS**	0.500	(-0.146) 0.847	0.059

**Fig 3:**
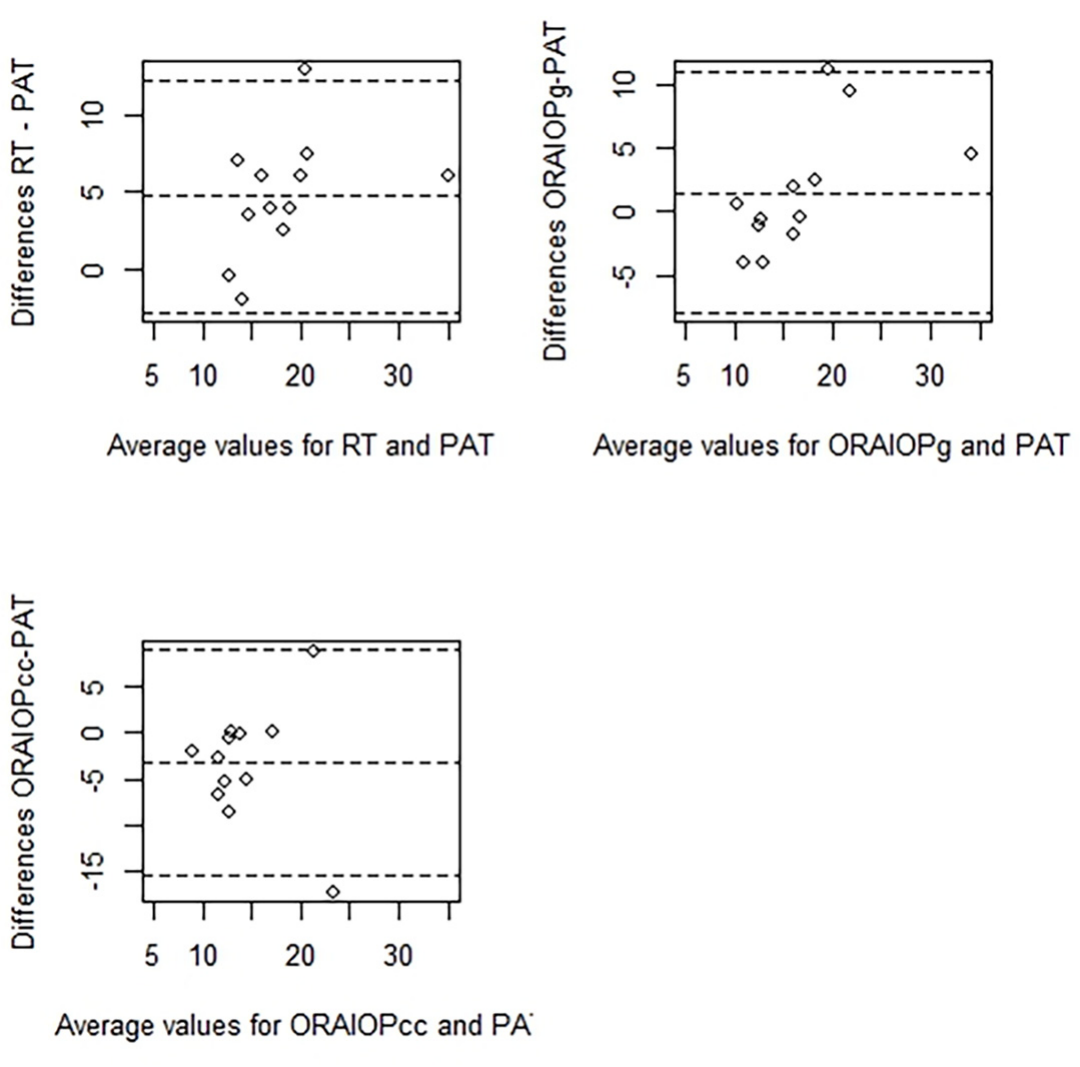
Bland-Altman plots show the agreement between rebound tonometry (RT-IOP), Goldmann-correlated intraocular pressure (ORA-IOPg), corneal-compensated intraocular pressure (ORA-IOPcc) and Perkins applanation tonometry (PAT-IOP) in right eyes.

**Fig 4:**
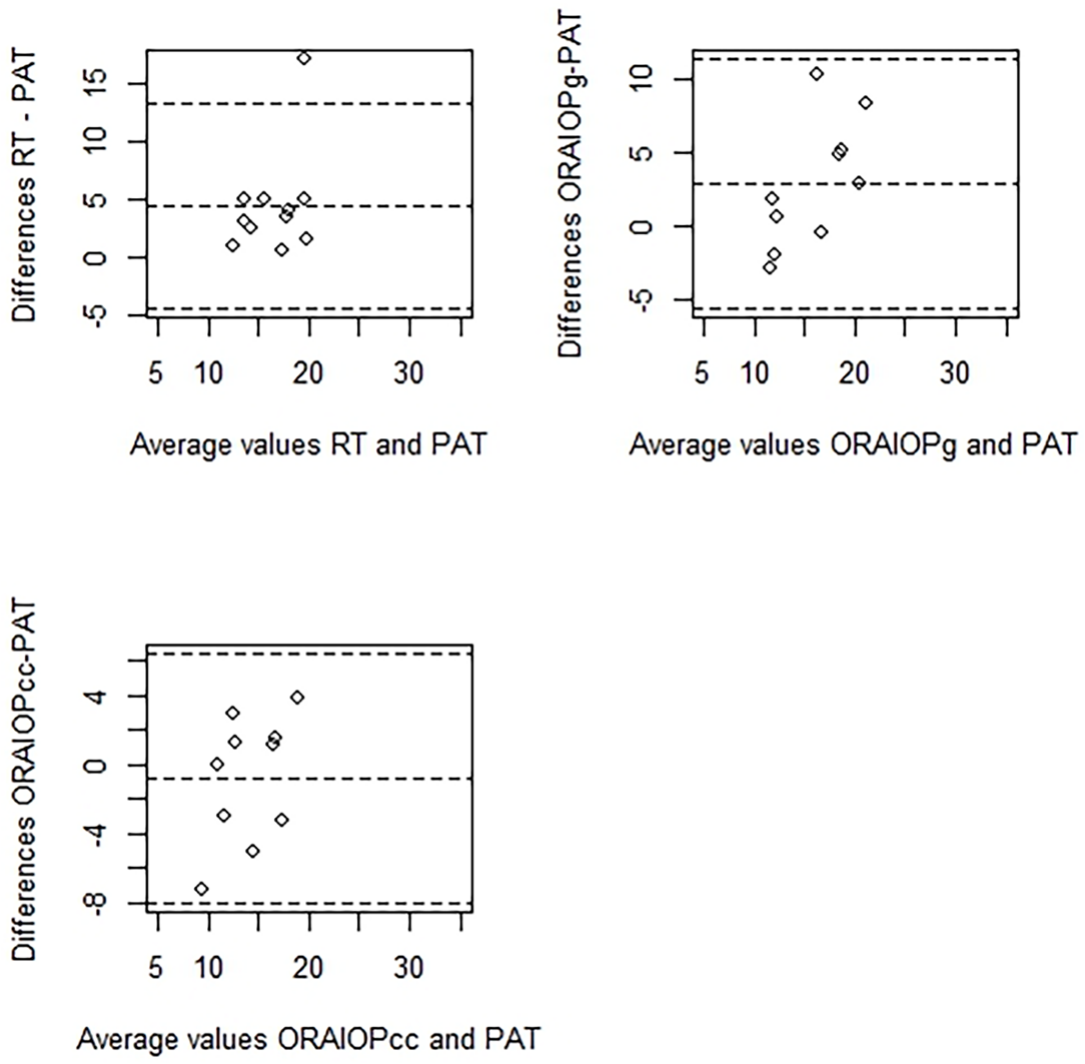
Bland-Altman plots show the agreement between rebound tonometry (RT-IOP), Goldmann-correlated intraocular pressure (ORA-IOPg), corneal-compensated intraocular pressure (ORA-IOPcc) and Perkins applanation tonometry (PAT-IOP) in left eyes.

Agreement between PAT and other tonometry methods (RT, IOPg and IOPcc) is presented as Bland-Altman plots in Figs 3 and 4.

**Fig 5:**
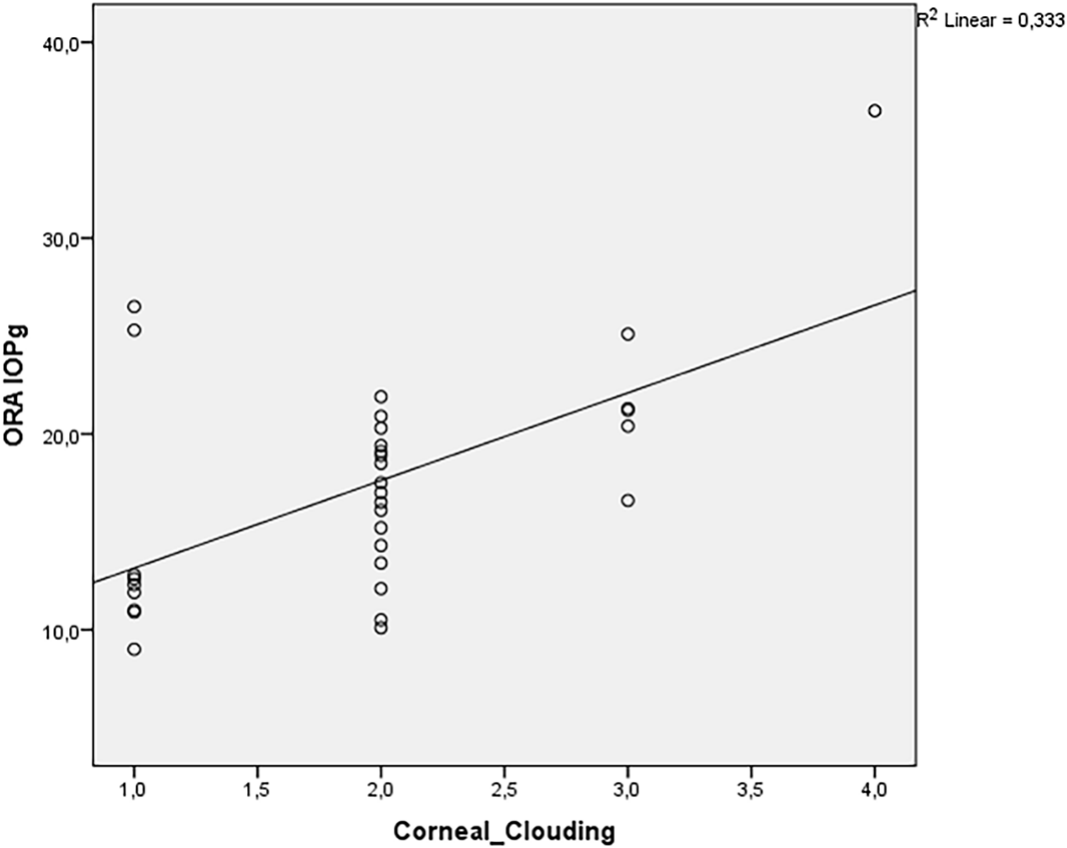
Regression graphs presenting Spearman´s correlation coefficient between Goldmann-correlated intraocular pressure (IOPg) and corneal clouding.

**Fig 6:**
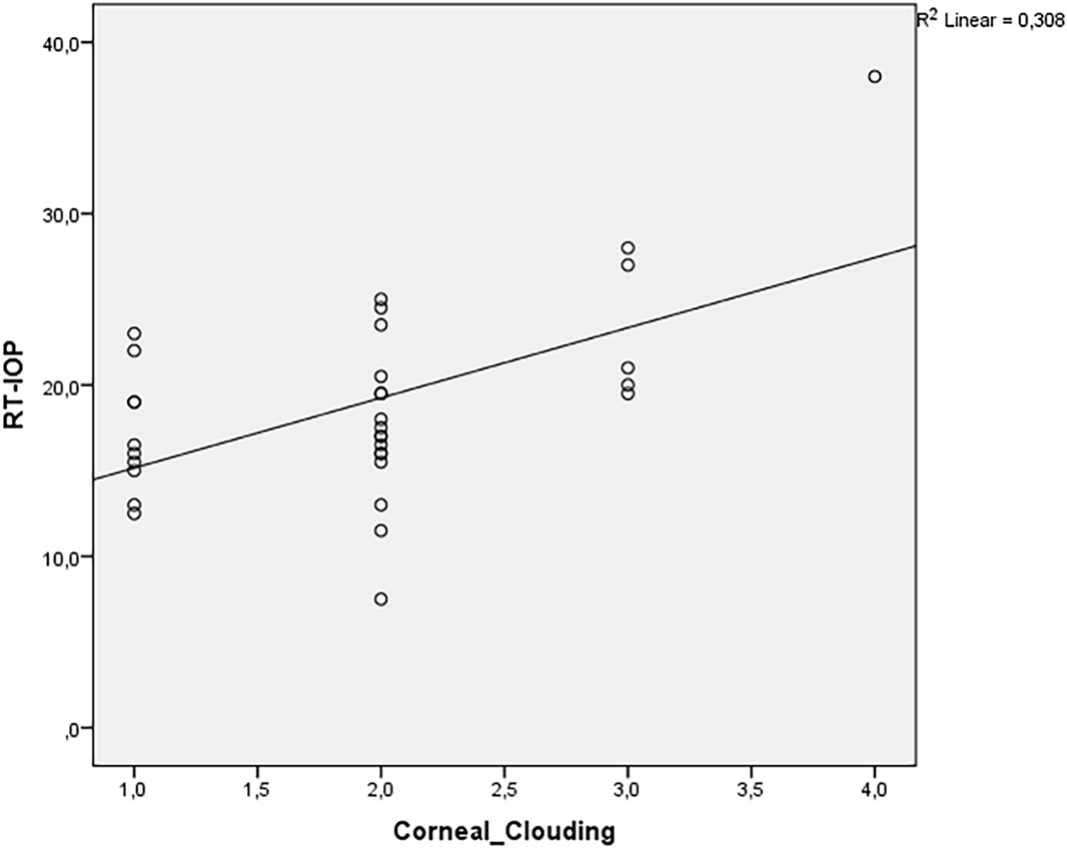
Regression graphs presenting Spearman´s correlation coefficient between rebound tonometry (RT) and corneal clouding.

**Fig 7:**
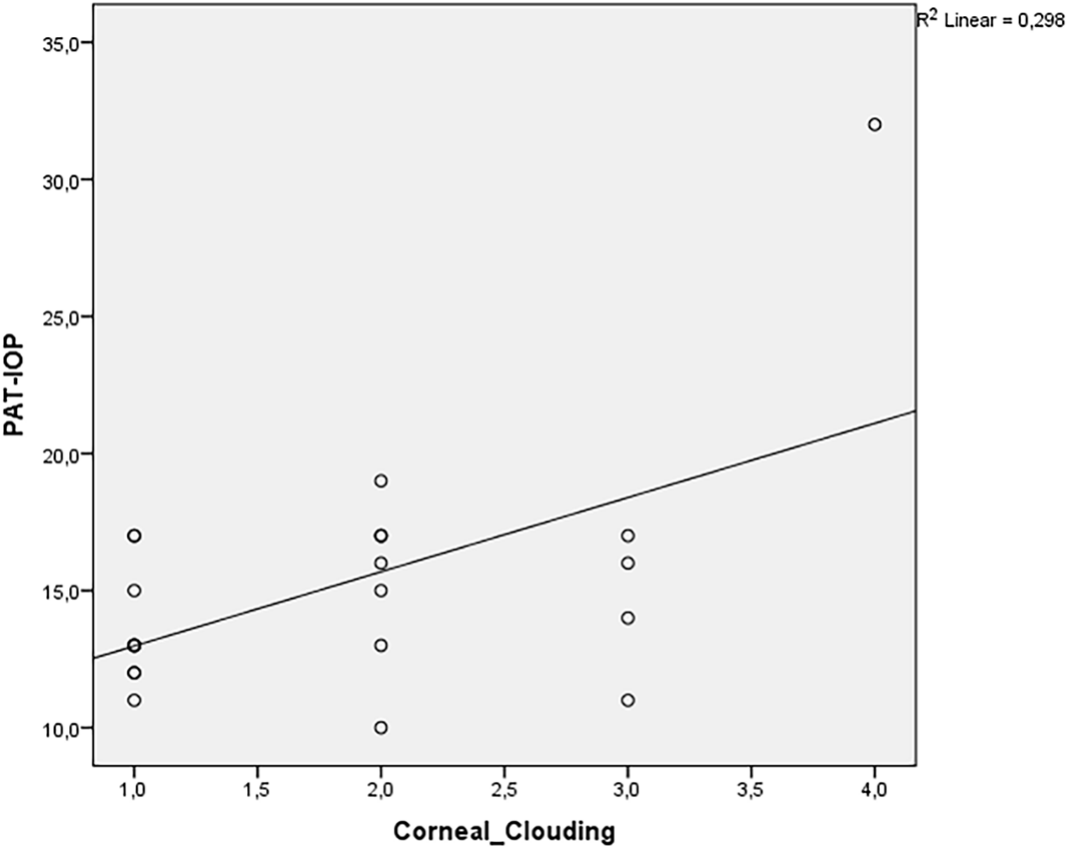
Regression graphs presenting Spearman´s correlation coefficient between Perkins applanation tonometer (PAT) and corneal clouding.

**Fig 8:**
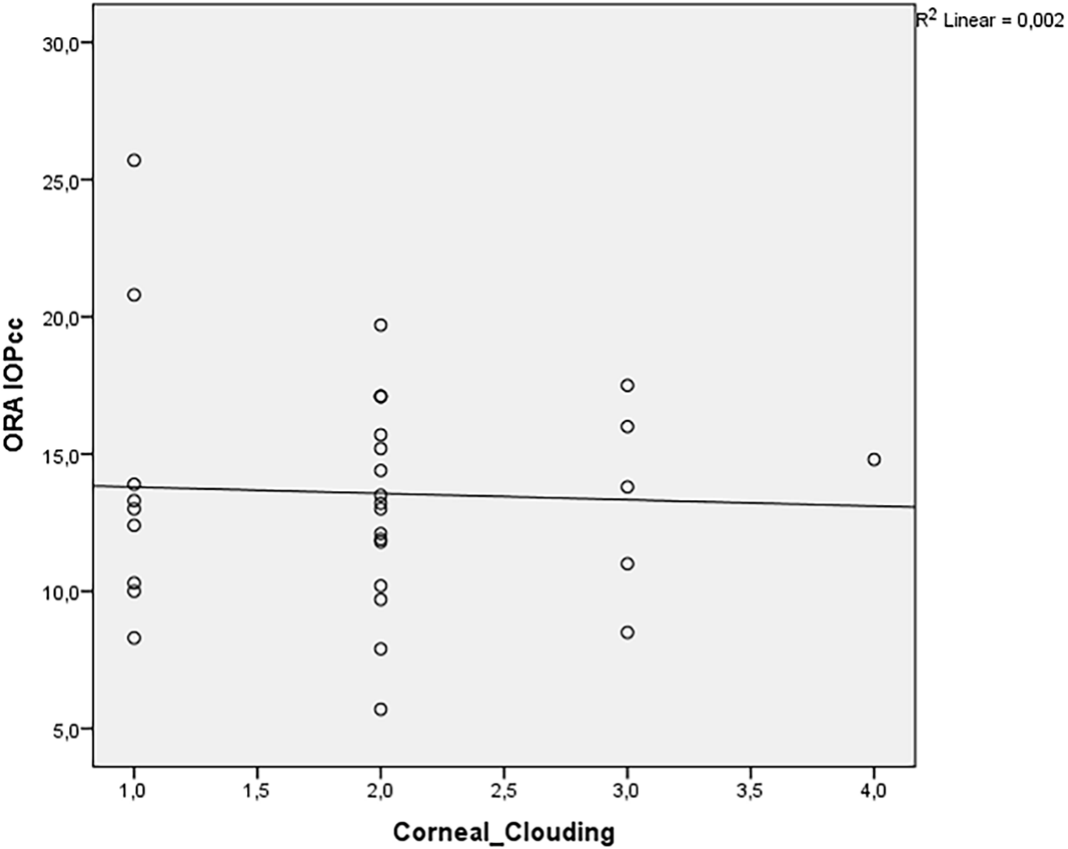
Regression graphs presenting Spearman´s correlation coefficient between corneal-compensated intraocular pressure (IOPcc) and corneal clouding.

Spearman´s correlation coefficient revealed a positive relationship between the degree of corneal clouding and CH, CRF, IOPg, RT-IOP and PAT-IOP in MPS patients. The relationship was strong for CH and CRF (correlation coefficients were 0.77 and 0.77, respectively), moderate for IOPg and RT-IOP (correlation coefficients were: 0.496 and 0.452, respectively) and weak for PAT-IOP (correlation coefficient of 0.352). There was almost no relationship between corneal clouding and IOPcc (correlation coefficient of 0.068). Figs 5–8 show scatterplots with linear regression lines.

## Discussion

This study was set to compare feasibility and tolerability of three different tonometry methods in MPS patients. Overall, RT and ORA were well tolerated by all study patients. In contrast, PAT was not accepted in almost one third of all MPS patients.

The median differences between applanation tonometry (PAT-IOP) and IOP measured with RT and ORA were statistically relevant in MPS. We observed an overestimating effect of RT compared to PAT, which is consistent with results reported for healthy subjects [13]. The median differences between ORA and PAT were smaller than discrepancies between RT and PAT and do not seem to be clinically relevant (mostly smaller than 2 mmHg). The median RT-PAT-difference was as high as 5 mmHg. This fact should be taken into account by interpretation of RT-IOP in MPS in clinical practice.

The degree of corneal clouding correlated strongly with CH and CRF. Our data show a trend, and lend support to the assumption that accumulation of GAG in the ocular tissue changes its biomechanical features and hence leads to increased CH and CRF values. Chui et al. showed a significant correlation between RT-IOP and CH and CRF but not between RT-IOP and central corneal thickness (CCT) in healthy subjects [14]. As RT-IOP was found to be significantly affected by CH and CRF, we can speculate that the relatively high CH and CRF values in our MPS patients, compared to the values reported in the literature for healthy subjects, may contribute to the overestimation of RT-IOP in comparison to PAT-IOP [15,16]. Our results are consistent with a study of Fahnehjelm et al., which reported increased CH and CRF in MPS [17]. Also the recent retrospective case-note review of patients suffering from MPS and glaucoma revealed that in patients for whom RT was used, mean IOP at diagnosis was approximately 10 mmHg higher than in patients for whom applanation tonometry was used [4]. The same study reported 2 patients, who were measured with RT and GAT at the same visit. In both cases RT-IOP were much higher than values of the applanation tonometry

We found a positive correlation between corneal clouding and IOPg, RT-IOP and PAT-IOP. However, there was almost no correlation between the grade of corneal clouding and IOPcc. IOPcc, unlike PAT-IOP, was not significantly correlated to corneal biomechanical features and probably represents “more real” IOP in GAG-affected eyes. These speculations need to be evaluated in prospective studies with healthy controls.

GAT and its hand-held alternative–PAT—are regarded to be the classical gold standard for IOP measurement. However, this method is dependent on a huge number of factors and error sources, inter alia: interaction between the tonometer head and precorneal tear film, concentration of fluorescein, corneal curvature and CCT, scleral rigidity, technique and experience of the examiner [18]. As histological preparations show that cornea and sclera of MPS patients are affected by the GAG-accumulation, it seems prudent to assume an additional tissue-related IOP-measuring error in MPS patients [19,20]. Furthermore, GAT, which requires correct positioning of patient and use of anesthetic eye drops, is not accepted even by some healthy subjects. For these reasons the acceptance of GAT in pediatric or disabled persons is very poor.

The well-known measurement error sources can be expected to be even greater in patients suffering from corneal disorders such as corneal clouding in storage diseases. Theoretically, a direct manometric measurement within the anterior chamber is superior to applanation tonometry, but of course it is not practicable in clinical routine [21]. However, GAT-IOP was found to differ significantly from intracameral IOP and the latter one was not correlated with CH and CRF in healthy subjects [22]. In the same study GAT-IOP was correlated stronger to CH and CRF than with CCT. This in consequence means for our study that it is likely, that the other tonometry devices (like ORA or RT) have been compared to a “weak gold standard”, as applanation tonometry may be much less reliable in MPS patients than in healthy subjects.

The limitation of our study was the lack of pachymetry data. Also the different number of eyes which were measured with different devices may bias the results.

To conclude, the excellent tolerability in paediatric and disabled patients (RT and ORA), portability (RT) and evaluation of biomechanical parameters (ORA) render both devices attractive alternative to applanation tonometry in MPS patients. Moreover, it seems to be important to use the same, individually adjusted and well-tolerated tonometry method for the follow-up of individual patients, as measurements with the different devices may vary considerably.
